# A Systematic Review of Wearable Sensor-Based Technologies for Fall Risk Assessment in Older Adults

**DOI:** 10.3390/s22186752

**Published:** 2022-09-07

**Authors:** Manting Chen, Hailiang Wang, Lisha Yu, Eric Hiu Kwong Yeung, Jiajia Luo, Kwok-Leung Tsui, Yang Zhao

**Affiliations:** 1 School of Public Health (Shenzhen), Sun Yat-sen University, Shenzhen 518000, China; 2 School of Design, The Hong Kong Polytechnic University, Hung Hom, Hong Kong, China; 3 Shenzhen Enstech Technology Co., Ltd., Shenzhen 518000, China; 4 Department of Physiotherapy, The University of Hong Kong-Shenzhen Hospital, Shenzhen 518000, China; 5 Grado Department of Industrial and Systems Engineering, Virginia Polytechnic Institute and State University, Blacksburg, VA 24061, USA

**Keywords:** fall risk assessment, sensor technology, community-dwelling older adults, functional test

## Abstract

Falls have been recognized as the major cause of accidental death and injury in people aged 65 and above. The timely prediction of fall risks can help identify older adults prone to falls and implement preventive interventions. Recent advancements in wearable sensor-based technologies and big data analysis have spurred the development of accurate, affordable, and easy-to-use approaches to fall risk assessment. The objective of this study was to systematically assess the current state of wearable sensor-based technologies for fall risk assessment among community-dwelling older adults. Twenty-five of 614 identified research articles were included in this review. A comprehensive comparison was conducted to evaluate these approaches from several perspectives. In general, these approaches provide an accurate and effective surrogate for fall risk assessment. The accuracy of fall risk prediction can be influenced by various factors such as sensor location, sensor type, features utilized, and data processing and modeling techniques. Features constructed from the raw signals are essential for predictive model development. However, more investigations are needed to identify distinct, clinically interpretable features and develop a general framework for fall risk assessment based on the integration of sensor technologies and data modeling.

## 1. Introduction

Accelerated population aging has dramatically increased the global healthcare burden [1]. The literature indicates that the global proportion of older adults will increase from 10% to 16%, with about one in every six individuals being 65 years or older, by 2050 [2]. Among the critical health consequences faced by the aging population, falls have been cited as a major cause of accidental death and injury [3]. It has been reported that approximately 33.3% of community-dwelling older adults experience at least one fall per year, with gait dysfunction having been identified as the main intrinsic cause [4]. Falls in older adults mainly result from aging mechanisms acting on both the sensorimotor and cognitive spheres. The structural and functional integrity of the peripheral sensory receptors and the musculoskeletal system deteriorate with age. Psychological affective factors such as depression, anxiety, and stress also speed up sensorimotor and cognitive decline [5]. This functional decline is notably characterized by an impaired control of balance, an altered gait pattern, reduced mobility, and falls [6]. Unlike in older adults, Cho, H. et al. [7] observed that the frequency of falls was positively associated with physical activity level, number of prescription medications, and being male in young adults. Physical activity increased fall-risk in young adults, but is generally protective of falls in older adults. Falls among the elderly are even more severe in developed countries and regions. In the United States, for example, it has been shown that 30–60% of community-dwelling older adults experience a fall every year, with more than 50% of them experiencing multiple falls [8]. Accidental falls have a significant impact on societies and healthcare system costs. The United States spends approximately $50 billion on fall-related medical costs annually, three-fourths of which are paid for by Medicare and Medicaid [9]. The negative health outcomes of falls can largely limit daily activities and induce “post-fall syndromes, such as dependence, loss of autonomy, immobilization, and depression in older adults, with hip fractures being the most common and severe outcome” [10]. Early warning and appropriate intervention via effective monitoring tools are crucial given the severity of the adverse consequences of falls [10,11].

Although the continuous monitoring of fall risk can help avoid the unnecessary deterioration of health, fall risk assessment is lacking in current practice due to several reasons. The functional tests used in clinical settings (Berg Balance Scale [BBS] [12], Tinetti Test [13], functional gait assessment [14], etc.) are effective measures of fall risk, but can be subjective and inconsistent [15,16] because they rely on the personal judgments of rehabilitation physicians or physiotherapists. In addition, long-term follow-ups of fall risk for large populations are costly in practice. The official website of the World Confederation for Physical Therapy has stated that there are only 1.4 rehabilitation professionals per 100,000 people in China, which is far below the international standard of more than 15 physiotherapists and 8–10 occupational therapists per 100,000 people [17]. The continuous assessment of fall risk requires intensive healthcare and clinical resources, but the currently limited professional resources are insufficient for the rapidly growing aging population. Therefore, accurate, easy-to-use, and affordable fall risk prediction approaches using advanced technologies are urgently needed [18].

In recent decades, sensor-based technologies for fall risk assessment have been widely explored. These technologies are split into three main categories: environmental perception techniques, optical motion capture systems, and wearable sensors. Gait assessment based on environmental perception techniques, such as force platforms and instrumented walkway mats, is usually carried out in gait laboratories equipped with complex testing equipment and analysis tools [15]. However, the high cost and operational difficulty make it difficult to extend this approach to large aging populations. Kinematic analysis based on optical motion capture systems such as time of flight, optoelectronic stereo photogrammetry, and Doppler radar can be easily affected by environmental dynamics. In addition, data processing for such technologies is highly complex and affected by user privacy issues [19]. Wearable sensors, as the name implies, are integrated into wearable objects or placed directly on the body, and range from pressure-sensing insoles to smart wrist bands that can monitor health and provide clinically relevant data for care [20]. Examples of wearable sensors include inertial measurement units (IMUs), pressure sensors, and electromyography (EMG) [21,22]. IMUs usually comprise accelerometers and gyroscopes, which are the most widely used sensors in fall-related studies [15,23,24]. Pressure sensors embedded in insoles or shoes can be used to measure plantar pressure and estimate gait speed [15]. EMG assessments, when combined with different functional tests, facilitate a comprehensive understanding of an individual’s gait and balance [16]. Therefore, wearable sensors offer an alternative approach to the efficient capture of kinematic data and may provide an easy-to-implement, objective method of fall risk assessment. Unlike non-wearable sensors, wearable sensors can continuously monitor gait with high accuracy [22], and such sensors are typically portable, small, and inexpensive, which makes them user-friendly and enables risk-monitoring in large aging populations.

**Figure 1:**
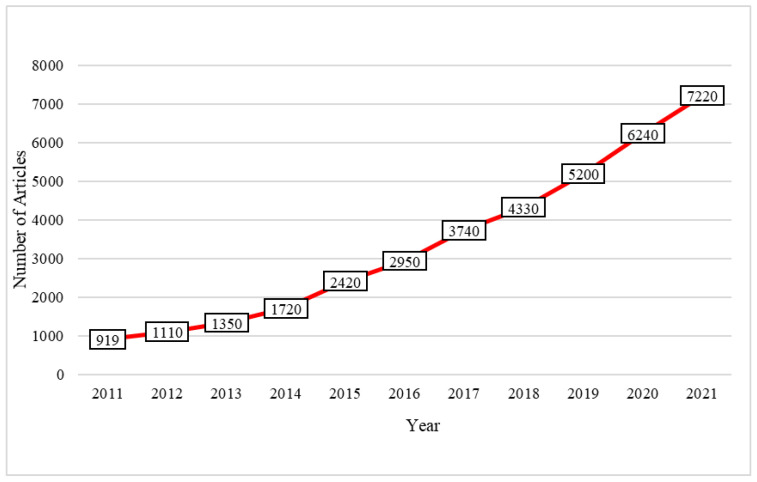
The number of articles on wearable sensor-based technologies for fall risk assessment in older adults published between 2011 and 2021, retrieved from Google Scholar.

To investigate public concerns about wearable sensor-based technologies for fall risk assessment in older adults, we retrieved recent relevant literature from Google Scholar using the search terms “fall risk assessment”, “wearable sensors”, and “elder”. As shown in Figure 1, there has been a growing interest in the use of wearable sensors to assess fall risk.

Some related studies have investigated the effectiveness of using wearable sensor data collected during functional tests to predict fall risk [25,26,27]. In these studies, machine learning methods were adopted to automatically identify fallers (F) and non-fallers (NF) based on features extracted from the sensor data. Specifically, the subjects were classified as either F or NF based on at least one of the following criteria: numerical assessment results of a standard functional test (e.g., BBS and Tinetti Gait and Balance Scale), self-reported fall occurrence within a follow-up period, or related hospitalization history. However, there was no consensus on the sampling frequency, sensor location, application scenario, and predictive models when predicting fall risk in older adults. Conversely, some wearable sensor-based fall risk assessment tools have recently become commercially available. One novel sensor for assessing fall risk in older adults is the FallSkip device [28]. This instrument applies a clinical protocol based on a modification of the TUG test, and uses inertial sensors to measure different variables (time, movement of the center of mass) in the different phases of the TUG. Another example is Mobility Lab™ (ML, APDM, Inc., Portland, OR, USA) [29], which is a system with six wirelessly synchronized IMUs [30]. The ML commercial software package allows the user to choose instrumented tests of balance and gait and automatically generates a report for each participant. The simplicity and automatic output enable simple data collection for clinical settings and/or large clinical trials [31]. However, little scientific research has been done to understand and quantify the impact and benefits these instruments may bring. In this study, we aimed to assess the current state of wearable sensor-based technologies that use predictive models or algorithms for fall risk prediction, with a focus on community-dwelling older adults. As there has not been any systematic review of the aforementioned challenge to fall risk assessment in community-dwelling older adults, we intended to fill this gap by analyzing the main technical aspects of recent studies and identifying an effective risk assessment approach that harnesses wearable sensor-based technologies. A systematic comparison was conducted from several perspectives such as sensor type, sensor placement, functional test, modeling method, and participant attributes. We also investigated the performance (in terms of accuracy, sensitivity, and specificity) of various fall risk assessment approaches involving different combinations of predictive models, sensors, and placements. Finally, in this paper, we discuss future trends in fall risk prediction via sensor technology and highlight several challenges encountered in the field.

The remainder of this paper is organized as follows: in the Methods section, we describe the search strategy, selection criteria, study selection, and data analysis of the selected articles; in the Results section, sensor-related information, functional tests, data processing and modeling, and the extracted response variables are described; the Discussion section describes modeling methods and the locations of sensors, and is followed by the Conclusion.

## 2. Methods

### 2.1. Search Strategy

**Figure 2:**
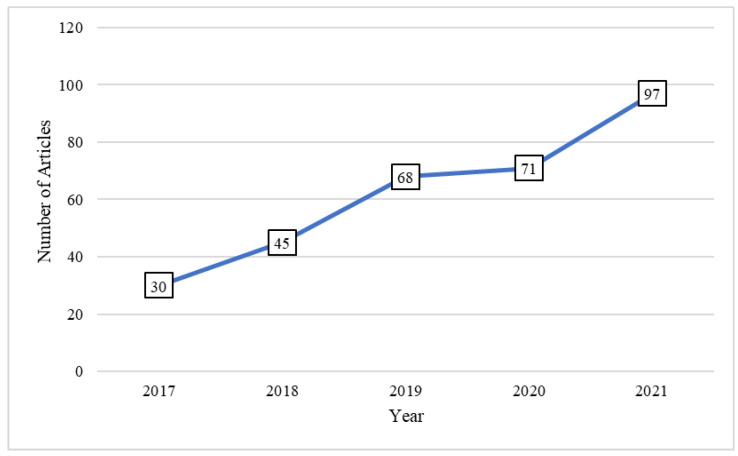
The number of relevant articles retrieved from PubMed, Scopus, and Web of Science between November 2017 and June 2022.

We searched three databases (PubMed, Scopus, and Web of Science) to identify potentially relevant articles between November 2017 and June 2022. Figure 2 shows the number of published articles in this period, indicating a rapid upward trend. The search terms were: (“accelerometer” OR “wearable sensors” OR “gyroscope” OR “magnetometers”) AND (“fall” OR “fall risk assessment”) AND (“gait analysis” OR “signal processing” OR “feature extraction”) AND (“aged” OR “geriatric” OR “gerontology” OR “senior” OR “elder” OR “old” OR “older adult”) AND (“general” OR “community-dwelling”). These search terms were validated by reviewing the retrieval of representative articles [32,33].

### 2.2. Selection Criteria

Articles were included if they met all of the following criteria: (1) the study population was older adults over the age of 65 or the technology was meant to be used on an aged population; (2) the fall risk assessment involved functional tests and not free daily-living activity tracking; (3) wearable sensors combined with functional tests were adopted for data collection; and (4) the full-texts of original papers published in peer-reviewed journals were available in English.

Articles were excluded from the review if they met one or more of the following criteria: (1) sensor data were collected during activities of daily living, (2) subjects were less than 65 years old, and (3) they were review studies, case studies, or commentary letters.

### 2.3. Study Selection

The selection process consisted of four stages: first, in the identification step, articles were identified via database search and manual inclusion; second, in the screening step, duplicates found in different databases were removed; third, in the eligibility step, articles were retained or removed based on inclusion and exclusion criteria, respectively; fourth, in the inclusion step, the remaining articles were analyzed in this review.

### 2.4. Data Extraction

To facilitate data analysis and better understand the current state of wearable sensor-based technologies that use predictive models for fall risk prediction, we extracted the following information from the selected articles: (1) Sensor-related information: information about the sensor types, sampling rates, and locations of sensors during the signal acquisition process were extracted and analyzed. (2) Functional test-related information: as data were acquired under different settings, it was necessary to determine which tests (functional) were combined with the sensors to best capture gait characteristics. (3) Data processing and modeling-related information: we investigated the data (pre)processing and analytical methods used to assess fall risk, which depended on the study objective and may have comprised statistical analyses of signal characteristics, classification tasks based on machine learning, and other approaches. (4) Outcome-related information: response variables based on the different tests and sensors, such as gait speed, stride length, total time duration, and sit-to-stand duration, were extracted.

## 3. Results

**Figure 3:**
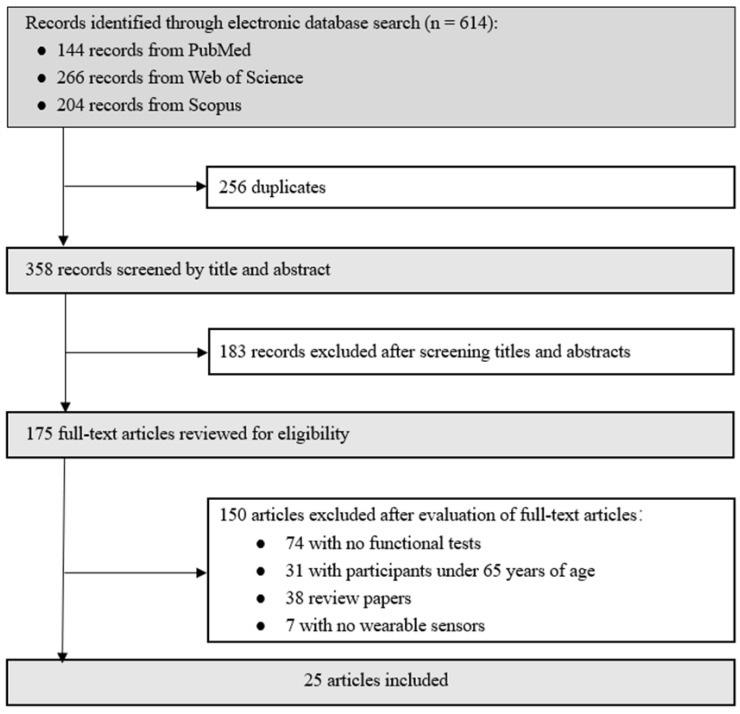
Flowchart of article selection process for the systematic review.

Figure 3 shows the literature search and review process. We retrieved 614 articles identified from PubMed, Scopus, and Web of Science. After the exclusion of 256 duplicate articles, 358 were screened by reviewing the titles and abstracts. Finally, 175 potentially relevant articles were reviewed in full-text, of which 25 met the inclusion criteria and were analyzed.

Table 1 summarizes the 25 articles included for further analysis, with the author and year of publication, characteristics of the study population, nature of the study, functional tests used, use of a sensor, and results listed. Specifically, response variables such as the number of falls and functional test scores were set as the ground truths for predictive model development. “Functional tests” refers to the type of functional tests undertaken by participants for fall risk assessment while wearing wearable sensors. “Number of sensors” refers to the number of wearable sensors used in the studies. “Feature engineering” indicates the process of constructing, extracting, and selecting features as inputs for the predictive models. “Model” refers to the modeling methods used for fall risk assessment.

### 3.1. Wearable Sensors

**Figure 4:**
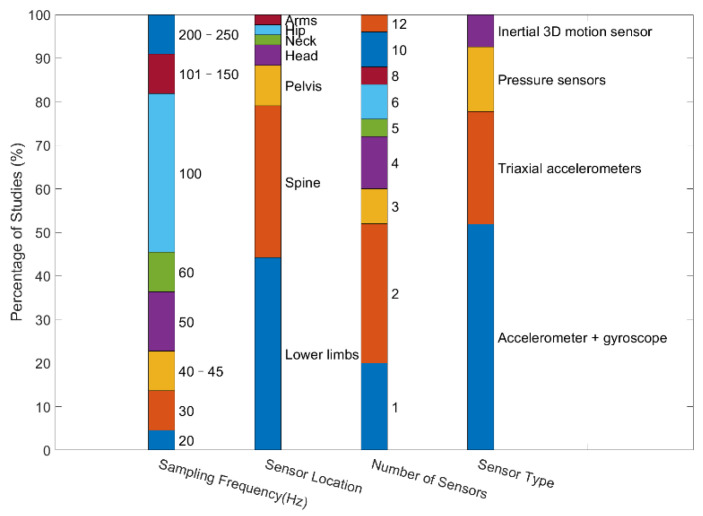
Summary of sensor information available in the reviewed studies.

Figure 4 summarizes the sensor information available in the 25 selected articles. Four types of sensors have been used: pressure sensors, accelerometers, gyroscopes, and inertial three-dimensional motion sensors. Specifically, 80% of the included studies used a combination of two or more sensors to capture motion signals. Fourteen studies used an IMU composed of an accelerometer and a gyroscope to capture motion signals, which appeared to be a common choice.

**Figure 5:**
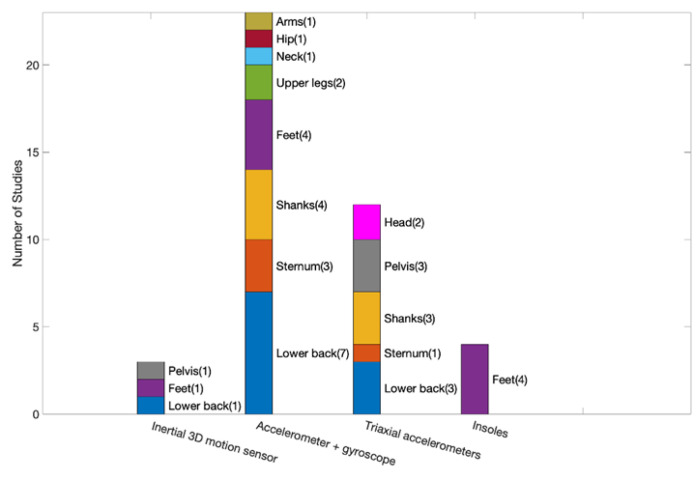
Association between sensor location and type.

In terms of sensor location, the spine (lower back, sternum) and lower limbs (feet, upper legs, shanks) were common body parts to which sensors were attached. To further examine the potential relationship between sensor locations and types, we summarize these two types of information in Figure 5. The IMU (accelerometer and gyroscope) was usually attached to the lower back, sternum, and shanks. The triaxial accelerometer was usually attached to the lower back, shanks, and pelvis. The spine region (lower back and sternum) was also chosen in 15 studies for quantifying center of mass and trunk movements. Nineteen studies placed sensors bilaterally on lower limbs (foot, shank, upper leg) to record spatial and temporal gait parameters, whereas four studies chose the pelvis. Some of the articles also reported spots that were less commonly used, such as the neck and chest. Several studies used a combination of sensors placed in different spots of the body. The sampling frequencies of the sensors used in these studies were diverse, ranging from 20 Hz to 250 Hz, with 100 Hz being the most common.

### 3.2. Functional Tests

Wearable sensors in combination with functional tests such as the timed up and go (TUG) test, sit-to-stand (STS) test, 4-stage balance test, and 6-min walk test (6MWT) can be employed as screening tools for fall risk assessment. In general, shorter time periods for the completion of these tasks indicated better gait patterns. Table 2 summarizes the frequency of the use of functional tests and their descriptions in the included studies. The TUG test (12 studies) was the primary assessment activity used, with some variants of it also used by a few (three) studies. The straight walking test (nine studies) and five time STS (5STS) test (three studies) were also commonly used for fall assessment. Only one study used standing tests and the alternate step test (AST) for assessment.

The TUG test is easy to implement, requires no special equipment or training, and can, therefore, be efficiently included as part of routine medical examinations [41]. However, the TUG test has limited ability to predict falls in community-dwelling older adults and should not be used in isolation to identify individuals at high risk of falls in this setting [54]. Compared with the standard TUG test, the environment adaptive TUG (EATUG) test has been reported to better depict balance and retain the characteristics of simplicity and high operability [41]. The 5STS has been widely adopted to examine lower extremity strength and determine an individual’s risk of falling [55]. All of these functional tests have certain environmental limitations. For example, the TUG test requires a straight-line distance of at least 3 m, which indicates a spatial requirement. Most of the aforementioned functional tests can be efficiently conducted by trained non-professionals and correlated to more sophisticated functional tests such as the BBS and Tinetti Gait and Balance Scale. These complex tests are usually held as the gold standard of fall risk assessment in clinical settings and must be conducted by healthcare professionals using various scales and scoring systems.

### 3.3. Data Processing and Feature Construction

Prior to data modeling and analysis, the raw signals received from the sensors were first processed by Butterworth low-pass filters to suppress possible noise in most of the included studies [44]. The processed data were further divided into segments based on motion via segmentation algorithms or manual inspection. For different data segments, the features were generated to characterize gait and balance in time and frequency domains.

Feature engineering methods, such as feature construction, extraction, and selection, are commonly used to generate features as inputs for certain statistical analysis or machine learning algorithms. Specifically, feature construction is used to discover relationships among features and augment the space of features by inferring or creating additional compound features. Thus, it can be used to expand the feature space [56], achieve data mining objectives, improve accuracy and comprehensibility, create truthful clusters, and reveal hidden patterns [57]. Feature extraction is used to extract a set of new features from the original features through functional mapping [58]. Feature selection is used to choose a subset of M features from the original set of N features (M≤N), such that the feature space is optimally reduced based on certain criteria [59]. Twenty of the selected studies adopted feature engineering for fall risk assessment. For the prediction of fall risk, the general process of feature construction comprised action recognition, original signal segmentation, and segmental feature extraction (including time sequence and spectrum features).

Two studies used deep learning methods (such as convolutional neural networks; CNNs) to predict fall risk, with the raw signal from sensors used as inputs for model development [38,52]. Three studies identified significant features (*p* < 0.05) using a statistical test [35,43,47]. They performed two-sample t-tests [35,43,47] and the Wilcoxon rank-sum test [43] on all the kinematics measured to find the significant ones, with statistical differences detected between the F and NF groups.

### 3.4. Predictive Method for Fall Risk Assessment

The development of predictive models based on sensor data was essential for fall risk assessment. Several predictive models were employed in the included articles, such as: logistic regression models, support vector machines (SVMs), CNNs, random forest, Lasso regression, elastic net, and long short-term memory. In general, the modeling approaches for fall risk assessment could be divided into two categories: (1) statistical or machine learning models that used features constructed from the sensor signal data as input and (2) deep learning methods that used the signal data as input. Both categories utilize the gait/balance scores from standard functional tests as output.

In terms of the model input, the included studies employed either gait features constructed from the raw sensor signal data or high-level spatiotemporal gait measures [51]. After feature construction, feature extraction or selection was used by some (seven studies) to reduce the number of features, make the model more general, and reduce over-fitting. After feature selection, machine learning models were established for identifying older adults at higher risk of falling, such as: decision trees (DTs) [45], relief-F [34], SVMs [34,35,41,48], Gaussian mixture models [37], logistic regressions [25,43,47,50,53], expectation maximization [37], minimum message length [37], random forest [33,40], and neural networks [26]. It should be noted that several models, such as elastic networks and Lasso regressions, enable synchronous feature selection. These methods also improve the interpretability of the selected features. Below, we introduce the main concepts of logistic regression and SVM, which were the most commonly used methods in the selected studies for fall risk assessment.

A logistic regression model can be applied to determine the odds of classification as F [60,61]:

(1) $$logit\;\varepsilon \left(Y\right)=\beta_{0}+\beta_{1}x\;$$

where Y is the Bernoulli-distributed response variable and x is the feature representing gait/balance. The β values are the linear parameters. The logit of the probability of success is then fitted to the predictors.

An SVM can be utilized to find the best separable plane with a certain level of error tolerance in the feature space [50]. It defines the decision boundary $w^{T}x+b=0$, and the class label of F or NF is represented as $y_{i}$∈ {−1, 1}. The classification is based on the rule $g(x)=sign(w_{i}^{T}x+b)$. In the classification problem, an SVM finds the best separable hyperplane determined by w and b to the following optimization problem.

(2) $$\underset{\left(w,b\right)}{\min}{\left\|w\right\|}_{2}\;s.t.\;y_{i}\left(w^{T}x_{i}+b\right)\geq \delta ,i=1,\cdots ,m$$

### 3.5. Features Used for Modeling

Different feature sets were used for modeling in the included articles, and there was no consensus on the significant features. Ruiz-Ruiz et al. proposed that the most significant parameters for frailty diagnosis were double-support time, gait speed, stride time, step time, and the number of steps/day or walking percentage/day [62]. In the case of fall risk detection, the kinematic parameters related to trunk stability or movements were the most relevant [62].

In the included articles, the significant features extracted by statistical analysis were: step length, gait velocity, total time duration, sit-to-stand duration, sit-to-stand lean angle, stand-to-sit duration, stand-to-sit lean angle, turn angle, turn peak velocity, turn duration, angular velocity, stand-to-sit jerk, maximum turning angular velocity, total duration of the test by IMU, total duration of rest periods, and postural transition duration [35,43,47]. The representative features extracted through feature engineering were: cadence, stride time variability, total walking time, gait speed, stride length, maximum angular velocity, maximum angular velocity radius, mean amplitude of mid-swing points, range of mid-swing point amplitude, variance, maximum acceleration, maximum velocity, peak power, maximum forward lean, variability domain, gait cycle time, and posterior deviation duration [26,40,41].

### 3.6. Evaluation Metrics

The metrics for evaluating the performance of fall risk assessment approaches were accuracy, sensitivity, specificity, receiver operator characteristic (ROC) curve, area under the ROC curve (AUC), and F1 score. Twenty articles analyzed the accuracy, sensitivity, specificity, and AUC. In terms of the reported results, the accuracy ranged from 0.57 to 0.90, the sensitivity ranged from 0.43 to 0.93, and the specificity ranged from 0.545 to 1.

## 4. Discussion

### 4.1. Participants

In this systematic review, we examined the literature on fall risk assessment in older adults, with a focus on the integration of sensor technology and data modeling, from 2017 to 2022. In terms of the study subjects, the most frequent sensor locations in the patient groups were the lower back and feet, whereas those in the general older adult groups were the lower back and shanks. The TUG test was the most commonly used functional test in the patient groups; although the same applied to the general elderly population groups, more complex tests were used as well.

### 4.2. Sensor Location

**Figure 6:**
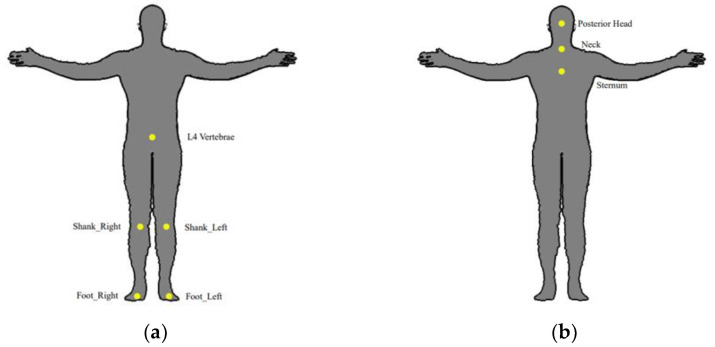
Recommended and not recommended sensor locations for data collection. (**a**) Recommended locations; (**b**) Not recommended locations.

The performance of fall risk assessment approaches can be affected by sensor locations. Figure 6 summarizes the recommended and not recommended sensor locations based on the included articles. Taking IMU as an example, Howcroft et al. identified that sensor data collected from regions near the center of mass, such as the lumbar and waist, were superior for identifying fall risk compared with other locations, which explained why a sensor placed on the lower back showed higher sensitivity than one placed on the sternum [63]. It should be noted that posterior head, neck, and sternum are not recommended for placing sensors based on the existing literatures. Howcroft et al. [26] reported that the predictive model based on a neural network and sensor data collected from head, pelvis, and left shank accelerometers achieved similar performance compared to the model with the data from pelvis only. They also identified the best model integrating SVM, ST assessment, and Relief-F feature selection without data collected from posterior head [34]. The fall risk assessment model with sensor data collected from the sternum region may result in poor performance on sensitivity. For example, low sensitivities of 48.10% and 56.0% were obtained by placing sensors on the sternum [25,43]. Although using deep learning-based method with the feature of angular velocity collected from the neck achieved a high sensitivity of 86%, the approach was not recommended given the fact that most participants feel uncomfortable when attaching the sensor to their neck [52].

### 4.3. Response Variables

The definition of “fall risk” has remained under debate as different measures have been adopted in the literature. Theoretically, the most reliable measure of true fall risk is prospective falls, recorded by continuously monitoring the subject to see whether he/she experiences a fall within a predefined period of time. However, prospective studies are usually difficult to conduct because they require intensive patient follow-ups and professional resources. Fall history, i.e., retrospective falls, can also be indicative of fall risk because previous falls have been identified to be strongly associated with increased fall risk [51]. However, compared with prospective studies, retrospective studies have a recall bias. Moreover, studies that use scales from functional tests as measures of fall risk, such as BBS, short form of BBS, and Tinetti Gait and Balance Scale, may fare better at detecting an individual’s current status.

### 4.4. Fall Risk Modelling Method

Statistical learning methods (such as logistic regression, SVM, and DT) have been widely applied for fall risk assessment. Logistic regression is particularly well-suited to studies in which the response is a dichotomous variable [64], although its performance in assessing fall risk varies across studies. SVMs allow kernel schemes to transform a feature space into any Hermitian space by a kernel function such as a polynomial kernel function or radial basis function; samples can then be clustered in the Hermitian space. SVMs have been proven to be applicable for predicting high-dimensional feature spaces as they regularize the coefficients of the separating hyperplane and can identify the best hyperplane that separates two clusters [50]. SVMs can thus achieve superior performance in fall risk prediction when the input feature space is high-dimensional and linearly inseparable [26,41]. Although DT models perform well in classifying F and NF without much computational power and can handle both continuous and categorical covariates [45], they are limited in terms of estimation tasks on small data, particularly when the response variable is a continuous attribute. Some studies have developed deep neural networks for modeling when dealing with large complex datasets [38].

Deep learning methods, such as convolutional neural networks (CNN) [38,49,52], use the raw inertial data directly as input. Deep learning allows for the creation of computational models that are composed of multiple processing layers and learn representations of data with multiple levels of abstraction [65]. This can result in more powerful models, because the complexity of the feature computations is dictated directly by the data and by the quality of the model predictions, rather than by the preconceptions of the operator [66] which can achieve high accuracy. However, it has some limitations for small samples.

### 4.5. Extracted Features

Identifying significant features and understanding their intrinsic associations have been recognized as the keys to the successful implementation of fall risk assessment approaches. Gait speed is calculated by dividing the total length by the walking duration. The jerk indicates the rate of change in acceleration. It has been reported that a shorter step length and faster gait speed are correlated to higher dynamic balance because the center of mass is closer to the moving base of support that confers stability [67]. Increased gait variability was found to be associated with falls in older adults and speculated to reflect a loss of automatic rhythm of gait [68]. In older adults, increased temporal gait variability has been linked to impaired physiological factors such as impaired postural control and proprioception, whereas cadence is thought to indicate the rhythm of gait [69]. Based on the fall risk assessment performance in the included studies, the predictive models developed from the features constructed from TUG achieved the highest sensitivity. For TUG and variants of the TUG test, the commonly constructed features were the angular velocity, TUG completion time, walking assistance, step length, gait velocity, sit-stand-sit jerk, and maximum turning angular velocity. For straight walk such as 6MWT, 7.62-m walk test, and 10-m walk test, the commonly constructed features were cadence, stride time, stance time, swing time, ML CoP path stance phase CoV, gait cycle time, gait speed, stance ratio, root mean square, and coefficient of variation. The total duration of the test by IMU, total duration of the rest periods, PT (postural transition) duration, PT peak angular velocity, and stand-sit jerk were the most commonly used features constructed from 5STS. However, most of the constructed features from functional tests are unlikely to be easily interpreted by non-professionals. Some features showed clinical significance such as gait speed, step length, gender, TUG completion time, walking assistance, step length, cadence, stride time, stance time, and swing time. Some features are not obviously interpretable, but can enhance prediction power, such as the angular velocity, sit-stand-sit jerk, maximum turning angular velocity, ML CoP path stance phase CoV, stance ratio, root mean square, coefficient of variation, PT peak angular velocity, RMS_ML(Walking), RMS_ML(StSi), RMS_V(SiSt), RMS_AP(StSi), Minimum_AP(SiSt), RMS_V(Walking), Maximum_V(SiSt), Range_ML(SiSt), and Ratio_Harmonic_ML.

### 4.6. Non-Wearable Sensors

Overall, wearable sensor-based technologies and data modeling methods offer accurate, affordable, and easy-to-use approaches for objective fall risk assessment. Compared to wearable sensors, non-wearable sensors have some limitations in large aging populations. For example, although recent advances in time of flight systems have been reported to increase the accuracy of identification of gait patterns to 84–94% [70], their reliability has not been validated for practical use [15]. Motion capturing systems such as Kinect-based systems may not “see” the risk when the camera’s field of view is obstructed [71]. Doppler radar is often used for fall detection and not fall risk assessment because it has the advantage of “seeing” through furniture. However, the lack of robust results for radar likely reflects the algorithmic challenges of using radar signals for reliable automated fall risk assessment. Radar’s inability to capture spatial parameters limits the number of fall risk measures that can be assessed [71]. Force platforms such as Wii balance boards and laser range finders are generally expensive, require dedicated laboratory environments and skilled technical personnel [21], and have low accuracy. Another alternative is GAIT Rite, which has a key shortcoming in gait assessment in that data capture is restricted to a few steps at a time. Therefore, GAIT Rite does not provide information regarding its longer-term variability on any gait measures [15].

## 5. Conclusions

Age is highly correlated with declines in physical, cognitive, and sensory functionalities. These functional impairments increase the chances of falling, which can have negative and fatal consequences. Fall risk assessment based on sensor technologies and data modeling methods has become a promising approach to the care of older adults. To date, a wide range of sensor technologies used in combination with data analytics tools have been examined. In general, these approaches provide an accurate and effective surrogate for fall risk assessment. Fall risk prediction can be influenced by various factors such as sensor location, sensor type, features utilized, and data processing and modeling techniques. Features constructed from raw signals are essential for predictive model development. However, the mixed effects of individual heterogeneity (such as age group and chronic disease) on the assessment are not clear as they vary across studies.

In practice, sensor-based data are usually noisy and contain unwanted vibration interfaces. The signal data of older adults can be even more complex, with unstable gaits making it difficult to find relevant harmonic ratios. Therefore, it is challenging to extract features that reveal the gait patterns of older adults. In addition, a gap remains between new technology and user acceptance among the elderly. More investigations are needed to identify distinct, clinically interpretable features and develop a general framework for fall risk assessment based on the integration of sensor technologies and data modeling.

## Figures and Tables

**Table 1:** Summary of the 25 included articles.

Author (Year)	Participant (Number, Age)	Response Variables	Functional Tests	Number of Sensors	Wearable Sensor Type	Sensor Location	Frequency (Hz)	Feature Engineering	Model	Accuracy	Specificity	Sensitivity
Sample et al. (2017) [25]	150 (74.35 ± 9.00; 91 NF, 59 F)	Retrospective	TUG ^1^	8	IMU ^2^ (accelerometer + gyroscope)	Spine (chest), spine (lower back), each foot	-	Y	Stepwise logistic regression	-	82.10%	48.10%
Howcroft et al. (2017) [26]	75 (75.2 ± 6.6; 47 NF, 28 F)	Prospective fall risk prediction	7.62 m under single- and dual-task conditions, 6MWT ^3^	6	Pressure-sensing insoles and triaxial accelerometers	Head, pelvis, left and right shanks, feet	50, 120	Y	Neural network	57%	65%	43%
Greene et al. (2017) [32]	422 (75.4)	1-year fall history	TUG ^1^	4	IMU ^2^ (accelerometer + gyroscope)	Shanks	102.4	Y	Regularized discriminant classifier	72.7%	54.50%	90.91%
Shahzad et al. (2017) [27]	23 (72.87 ± 8)	BBS ^17^	DR ^4^ tasks twice (TUG ^1^, FTSS ^5^, AST ^6^)	1	Triaxial accelerometer	Spine (lower back)	41	Y	Lasso regression	-	-	-
Drover et al. (2017) [33]	76 (74.15 ± 7.0)	6-month follow-up prospective fall	6MWT ^3^	3	Triaxial accelerometer	Posterior pelvis, left and right lateral shanks	50	Y	Random forest	77.30%	84.70%	66.10%
Howcroft et al. (2018) [34]	75 (75.2 ± 6.6)	6-month follow-up prospective fall	7.62-m walk	5	Pressure-sensing insoles, triaxial accelerometer	Insole, left shank, pelvis, head	100	Y	Relief-F, SVM ^13^	94.40%	100.00%	85.70%
HaiQiu et al. (2018) [35]	196	Retrospective	SIT ^7^, LOS ^8^, 5STS ^9^, MF ^10^, CRT ^11^, FES ^12^, 3-m TUG ^1^	10	IMU ^2^ (accelerometer + gyroscope)	Spine (low back), upper and lower legs	100	Y	SVM ^13^	89.40%	84.90%	92.70%
Hellmers et al. (2018) [36]	157 (75.22 ± 3.83)	SPPB ^14^, SCPT ^15^, 6MWT ^3^, frailty criteria, counter movement lump	aTUG ^27^	2	IMU ^2^ (accelerometer + gyroscope)	Hip	100	Y	Hierarchical classification model	96%	-	-
Ghahramani et al. (2019) [37]	86 (80.4 ± 7.9)	Fall history	Five common standing tests, BBS ^17^	1	Inertial 3D motion sensor (MTw from Xsens technology)	Pelvis	50	Y	GMM ^18^, EM ^19^, MML ^20^	-	75.7% and 77.7% ^34^	78.6% and 82.1% ^34^
Buisseret et al. (2020) [38]	73 (>65)	6-month follow-up prospective fall	TUG ^1^, 6MWT ^3^	2	IMU ^2^ (accelerometer + gyroscope)	Spine (lower back)	100	N	CNN ^21^	76%	-	-
Yu et al. (2021) [39]	85 (69–105)	SFBBS ^31^	TUG ^1^	1	Triaxial accelerometer	Spine (lower back)	45	Y	Lasso regression	-	79%	74%
Lockhart et al. (2021) [40]	171 (74.3 ± 7.6)	6-month follow-up prospective fall	10-m walking test	1	Triaxial accelerometer	Spine (sternum)	100	Y	PCA ^22^, Random Forest predictive model	81.6 ± 0.7%	80.3 ± 0.2%	86.7 ± 0.5%
Diao et al. (2021) [41]	103	Questionnaires, BBS ^17^	EATUG ^23^	4	IMU ^2^ (accelerometer + gyroscope)	Shanks (15 cm below knee joint)	60	Y	SVM ^13^	90.50%	92.90%	85.70%
Choi et al. (2021) [42]	37 (69.6 ± 4.3)	3-m TUG ^1^	Walking a circular sidewalk route for 3 min	3	Inertial 3D motion sensor (MTw from Xsens technology)	Pelvis and feet	60	Y	Ridge regression	-	-	-
Atrsaei et al. (2021) [43]	458	12-month follow-up prospective fall	5STS ^9^	2	IMU ^2^ (accelerometer + gyroscope)	Spine (sternum)	200	Y	Logistic regression	-	69%	56%
Bet et al. (2021) [44]	74	12-month follow-up prospective fall	Variants of TUG ^1^: TUG-S ^24^, TUG-M ^25^, TUG-D ^26^	1	Triaxial accelerometer	Spine (waist)	100	Y	N (Shapiro-Wilk normality test)	75%	76%	71%
Song et al. (2022) [45]	48	BBS ^17^	20-m long walk for over 2 min	2	Pressure sensors	Insole	20	Y	DT ^28^, GBDT ^30^, AdaBoost	87.5%	75%	100%
Wu et al. (2022) [46]	48 (74.5 ± 6.7)	BBS ^17^, TUG ^1^, fall history	Walk for at least 2 min	2	Pressure sensors	Insole	-	N	MhNet	73.27%	70.4%	76.72%
Lin et al. (2020) [47]	51 PD ^29^ patients (65.7 ± 8.4)	6-month follow-up prospective fall	7-m TUG ^1^	12	IMU ^2^ (accelerometer + gyroscope)	Feet, spine (trunk), spine (sternum), arms	-	Y	Binary logistic regression	-	78.40%	71.40%
Polus et al. (2021) [48]	72 patients following total hip arthroplasty (71.87 ± 6.45)	TUG ^1^	TUG ^1^	10	IMU ^2^ (accelerometer + gyroscope) + iPod touch (3D ^16^ gyroscope + MEMS ^32^ accelerometer)	Above and below each knee	-	Y	PCA ^22^, SVM ^13^	90%	59%	93%
Xiaomao et al. (2021) [49]	105 stroke survivors (56 ± 14)	SFBBS ^31^	3-m TUG ^1^	2	IMU ^2^ (accelerometer + gyroscope)	Spine (back trunk)	30	Y	Siamese network	85% ± 6%	-	-
Yu-Cheng et al. (2020) [50]	50 post-stroke patients (57.4 ± 14.13)	SFBBS ^31^	3-m TUG ^1^	2	IMU ^2^ (accelerometer + gyroscope)	Spine (L4 vertebrae)	30	Y	Elastic net, logistic regression	84%	94%	64% ± 5%
Tunca et al. (2020) [51]	76 neurological disorder patients (76.8 ± 10.3)	12-month fall history	Walk back and forth along an 8-m straight line	4	IMU ^2^ (accelerometer + gyroscope)	Dorsum of both feet	100	Y	LSTM ^33^ (gait parameters as input)	92.10%	-	-
Roshdibenam et al. (2021) [52]	100 patients with different mental or physical impairments (65–96)	TUG ^1^, 30-s stand, 4-stage balance tests, measurement of orthostatic blood pressure, clinicians’ observations, and SIB score	TUG ^1^	6	Run Scribe IMU ^2^ pods (accelerometer + gyroscope)	Right and left feet and neck	250	N	CNN ^21^	71%	55%	89%
Dierick, F et al. (2022) [53]	73 patients with different mental or physical impairments	6-month follow-up prospective fall	TUG ^1^	2	IMU ^2^ (accelerometer + gyroscope)	Spine (L4 vertebrae)	100	Y	Multiple logistic regressions	-	95.9%	29.2%

^1^ TUG: time up and go; ^2^ IMU: inertial measurement unit; ^3^ 6MWT: 6-min walk test; ^4^ DR: directed routine; ^5^ FTSS: five time sit-to-stand; ^6^ AST: alternate step test; ^7^ SIT: sensory integration test; ^8^ LOS: limits of Stability; ^9^ 5STS: five time sit-to-stand; ^10^ MF: motor Function; ^11^ CRT: choice reaction test; ^12^ FES: computerized falls efficacy scale; ^13^ SVM: support vector machine; ^14^ SPPB: short physical performance battery; ^15^ SCPT: stair climb power test; ^16^ 3D: three-dimensional; ^17^ BBS: Berg Balance Scale; ^18^ GMM: Gaussian mixture model; ^19^ EM: expectation maximization; ^20^ MML: minimum message length; ^21^ CNN: convolutional neural network; ^22^ PCA: principal component analysis; ^23^ EATUG: environment adaptive TUG; ^24^ TUG-S: simple configuration of the TUG; ^25^ TUG-M: motor-task TUG; ^26^ TUG-D: dual-task TUG; ^27^ aTUG: ambient TUG; ^28^ DT: decision tree; ^29^ PD: Parkinson’s disease; ^30^ GBDT: gradient boosting decision tree; ^31^ SFBBS: short form of Berg Balance Scale; ^32^ MEMS: micro electro mechanical systems; ^33^ LSTM: long short-term memory. ^34^ The standing with feet together and standing with one foot in front, sway index distinguished older fallers from non-fallers with specificity of 75.7% and 77.7%, respectively, and sensitivity of 78.6% and 82.1%, respectively.

**Table 2:** Frequency of use of functional tests and their descriptions.

Functional Test	Frequency	Description
TUG ^1^	12	Time in seconds taken by the individual to get up from a chair without support, walk straight for 3 m, turn, walk back the 3 m, and sit in the chair without support.
Variants of the TUG test: TUG-M ^2^, TUG-D ^3^, aTUG, EATUG ^4^	3	TUG-M: the individual must carry a glass full of water. TUG-D: the individual must perform both a motor and a cognitive task; the motor task is to transfer coins between two pockets of a lab coat and the cognitive task is to calculate successive subtractions of 7, starting from 100, out loud. aTUG: The aTUG system is used for automated TUG tests and includes force sensors (FS) in each chair leg, a laser range scanner (LRS) and a light barrier (LB). EATUG: the individual must bypass and overpass an obstacle, such as ascending and descending stairs.
Straight walk	9	6MWT ^5^: the distance walked in 6 min to the nearest meter is measured; the individual must walk back and forth along a straight line approximately 8 m long; 7.62-m walk test; and 10-m walk test.
Five common standing tests	1	The individual must: (1) stand with eyes open for 2 min; (2) stand quietly with eyes closed for 30 s; (3) stand on one foot for 10 s without support; (4) stand with feet together for 1 min; and (5) stand with one foot in front of the other with the heel of the forward foot touching the toes of the other foot for 1 min.
5STS ^6^	3	The total duration to perform postural transitions (sit/stand), traditionally measured by a stopwatch, is used to discriminate between patients with and without balance disorders.
AST ^7^	1	The individual must place the whole of each foot, alternatively and rapidly, on and off of a platform (19 cm high and 40 cm wide).
Walking a circular sidewalk route for 3 min	1	The individual must walk a circular sidewalk route for 3 min.

TUG ^1^: time up and go; TUG-M ^2^: motor-task TUG; TUG-D ^3^: dual-task TUG; EATUG ^4^: environment adaptive TUG; 6MWT ^5^: 6-min walk test; 5STS ^6^: five time sit-to-stand; AST ^7^: alternative step test.

## Data Availability

Not applicable.
